# Patient Selection for Epicardial Ablation—Part II: The Epicardial Approach and Current Challenges Associated with Epicardial Ablation

**DOI:** 10.19102/icrm.2019.101105

**Published:** 2019-11-15

**Authors:** Justin A. Edward, Duy T. Nguyen

**Affiliations:** ^1^Section of Cardiac Electrophysiology, Division of Cardiology, University of Colorado Denver, Aurora, CO, USA

**Keywords:** Arrhythmia, catheter, epicardial, radiofrequency ablation

## Abstract

Since their inception, percutaneous epicardial approaches have become increasingly common in clinical practice with the advent of new technology and the growth of catheter ablation for both ventricular and supraventricular arrhythmias. In addition to identifying the arrhythmogenic foci, there remain challenges to successful epicardial ablation such as the choice of energy source, optimizing irrigation during ablation, and anatomic barriers such as epicardial fat and coronary vessels. The performance of continued translational studies to understand how each of these factors contribute to lesion formation will be essential to guide future advances in the field of epicardial ablation.

## Introduction

**Figure 1:**
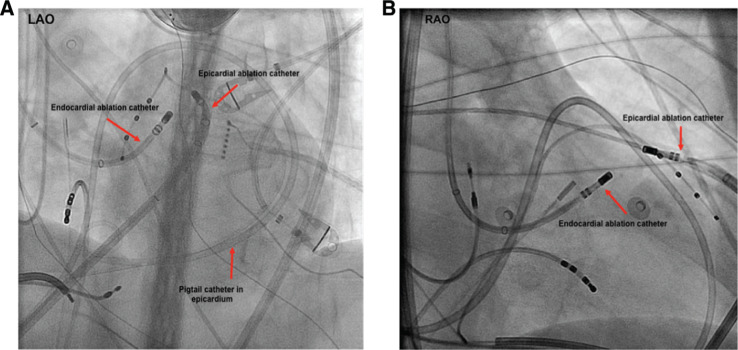
Endocardial–epicardial bipolar ablation configuration. Bipolar ablation across an LV summit midmyocardial circuit may require epicardial access for one of the ablation poles. **A and B:** Fluoroscopic views (anteroposterior) of two ablation catheters in a bipolar configuration, targeting the LV summit area for this patient. LAO: left anterior oblique; RAO: right anterior oblique.

Traditional epicardial catheter ablation was first employed in patients with Chagas disease with ventricular tachycardia (VT) and an epicardial substrate.^1^ In more recent years, this method has grown dramatically, and modifications to the epicardial approach such as the “needle-in-needle” technique have been made.^2^ Methods to improve the safety of epicardial catheter ablation are of interest as both significant pericardial bleeding (up to 10% of cases) and pericarditis are common complications attributed to epicardial access.^2,3^ Epicardial access is occasionally used to treat supraventricular tachycardias, including accessory pathways, atrial fibrillation (AF), and inappropriate sinus tachycardia following failed endocardial ablation **(Figure 1)**.^4–6^

Epicardial ablation is primarily limited to deployment in high-volume, tertiary ablation centers for the treatment of patients who have previously failed the endocardial approach. In our center, we commonly employ endocardial–epicardial bipolar ablation to target midmyocardial VT circuits. Given the developments that have occurred over the last two decades, epicardial access is now established as an effective strategy for epicardial VT ablation and epicardial strategies are being further employed for various supraventricular arrhythmias, including AF. In a 2010 study of three tertiary ablation centers in both Europe and the United States, approximately one in five VT ablations utilized epicardial mapping and/or catheter ablation.^3^ This paper aims to review the indications, techniques, and developments in the field of epicardial catheter ablation.

## The epicardial approach and potential considerations

The current standard of care for epicardial ablation is via the subxiphoid percutaneous technique. The traditional method of a subxiphoid percutaneous approach was first described by Sosa et al. in their report of epicardial VT ablation.^1^ Using a 6-in, 17-gauge Touhy epidural needle, which has a blunt tip, one can access the epidural space while minimizing the risk of vascular or myocardial injury.^3^ During this procedure, with fluoroscopic guidance, the needle is inserted at a shallow angle (< 30°) along the left border of the subxiphoid process and advanced toward the left shoulder for an anterior approach. Contrast is injected to visualize tenting of the parietal pericardium at the tip of the needle as it is advanced into the pericardial space. Once in the space, a guidewire, followed by a sheath placed over the wire (once the guidewire is confirmed to be in the pericardial space), is advanced into the pericardial space. Mapping or ablation catheters can then be positioned in the pericardial space via an introducer sheath.

**Figure 2:**
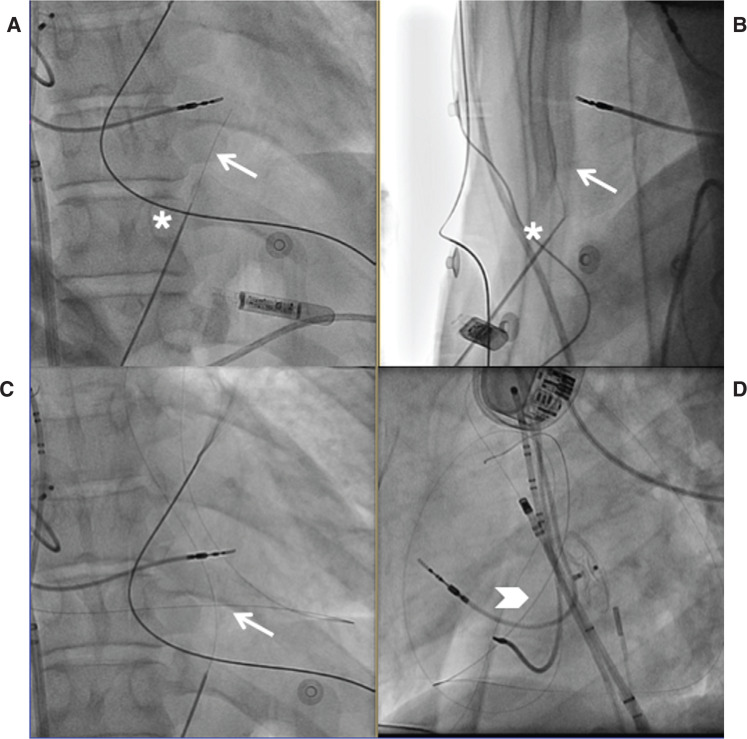
Epicardial access—“needle-in-needle” approach. In this stepwise image, an 18-gauge needle (Cook Medical, Bloomington, IN, USA) was first inserted for stability, through which the 21-gauge micropuncture is inserted (asterisk), as seen in the anteroposterior **(A)** and left lateral **(B)** projections. After entry was gained into the epicardial space using the micropuncture needle, an 0.18-in guidewire (arrow) was then inserted and epicardial placement was confirmed once the wire hugged the borders of the cardiac silhouette **(C)**. Both needles were removed and a micropuncture dilator was advanced into the pericardial space. An 0.35-in guidewire was placed through the dilator, and the wire was confirmed to be crossing multiple cardiac chambers and again wrapping around the borders of the cardiac silhouette in the left anterior oblique projection **(D)**.

Recent advances have been made resulting in a “needle-in-needle” approach **(Figure 2)** that employs a shorter (7-cm) 18-gauge needle beneath the sternum.^2^ This short needle provides stability and more tactile feedback for a longer (15- or 20-cm) 21-gauge needle to be inserted through the already placed 18-gauge needle. A guidewire is advanced via fluoroscopy and both needles are removed. Ultimately, dilators are used to eventually introduce an 8-French sheath into the pericardial space. When comparing the “needle-in-needle” technique directly to the Sosa technique, successful epicardial access was achieved in 100% of the 23 “needle-in-needle” cases as compared with in 94% of the 316 retrospective cases performed utilizing the Sosa technique. Of note, no differences were observed regarding major pericardial bleeding.^2^ In a multicenter observational study evaluating the performance of micropuncture against that of a larger bore needle, there was no significant difference in the incidence of inadvertent puncture of the myocardium between the two needle approaches. However, there was a significantly higher rate of large pericardial effusions and bleeding, with the larger bore needle requiring either drainage or open-heart surgery.^7^

To avoid serious bleeding complications during epicardial ablation, anticoagulants and antiplatelet agents should be discontinued prior to attempting access so as to minimize the risk of bleeding, and each patient should be typed and screened in case blood products need to be administered. A detailed medical and surgical history should be gathered before a patient undergoes a percutaneous epicardial approach. A history of prior cardiothoracic surgery, prior epicardial ablation, and/or prior pericarditis may increase the risk of pericardial adhesions, which can complicate the attainment of proper access. In patients with pericardial adhesions, limited surgical thoracotomy may be indicated, which allows for manual lysis of adhesions prior to visualization of the epicardial surface.^8^ Given the potential for injury to cardiac, thoracic, and abdominal structures that lie within this space, this surgical technique can help to minimize potential complications associated with conventional percutaneous access in patients with a history of cardiac surgery.

While up to one-third of patients may develop uncomplicated postprocedural pericarditis,^9^ the risk of serious complications such as hemopericardium and coronary or phrenic nerve injury can be reduced through careful planning. Precautions should be undertaken to mitigate the risk of procedural complications when performing epicardial ablation, including preablation coronary angiography to visualize potential major epicardial vessels that may be in close proximity with ablation lesions and high output pacing of the phrenic nerve to prevent collateral injury.^10,11^ If an ablation target is close to the phrenic nerve, air and saline can be instilled into the pericardial space to help prevent phrenic nerve injury.^12^ Ultimately, knowledge of the patient’s prior cardiac surgery and coronary anatomy are helpful in the avoiding potential procedural complications.

The most common adverse event following epicardial ablation is acute postprocedure pericarditis. The proposed mechanism behind this complication is likely attributed to an inflammatory response triggered initially by needle puncture through myopericardial tissue followed by wire and catheter manipulation in the pericardial space as well as ablation of the tissue itself. Symptoms of pericarditis can develop in up to 30% of patients following epicardial ablation,^13^ although most cases are self-limiting and respond to nonsteroidal anti-inflammatory medications (NSAIDs). NSAIDs or colchicine should be given to all patients postprocedurally for one to two weeks, with tapering initiated once patient symptoms improve. Additionally, the injection of glucocorticoids into the pericardial space, which has been shown to reduce pericarditis in animal models,^14^ is the standard of care following epicardial ablation to minimize postprocedure pericarditis. Direct administration of intrapericardial steroids and the use of NSAIDs have been effective in our center in reducing postablation pericarditis.

## Current challenges in epicardial ablation

The biophysical variables that predict effective epicardial ablation are similar to those used for endocardial ablation; however, there are fundamental differences between these two ablation strategies. The pericardial space provides a unique challenge for epicardial ablation due to the presence of epicardial fat and intrapericardial fluid **(Figure 2)**. Furthermore, endocardial blood flow, which provides convective cooling, is not present in the epicardium.

### Energy source

**Figure 3:**
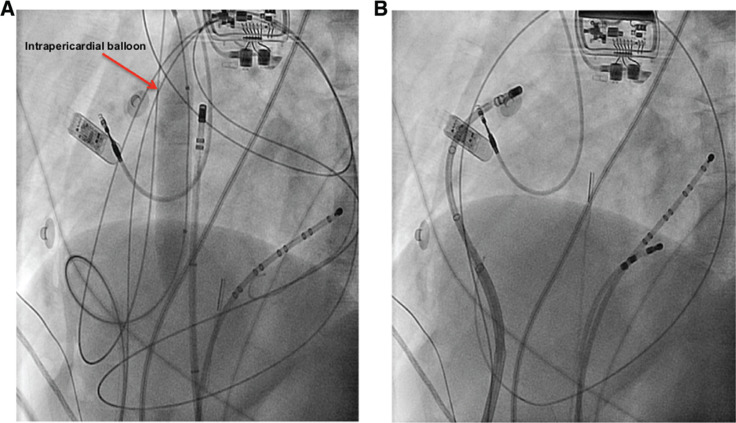
Displacement of the phrenic nerve by higher-impedance pericardial fluid to facilitate lesion formation. **A:** Fluoroscopy (anteroposterior view) of a pericardial balloon inflated at the level of the phrenic nerve to displace it and allow for endocardial ablation of the sinus node; however, in this case, endocardial ablation did not successfully suppress the inappropriate sinus tachycardia. We decided to ablate the epicardial sinus node inputs but were unable to reach the epicardial area of interest because of the intrapericardial balloon that was being used to move the phrenic nerve away. **B:** As such, we decided to infuse intrapericardial fluid to move the phrenic nerve away. However, normal saline led to poor lesion formation and we instead used a high-impedance fluid of D5W to allow for adequate ablation lesion formation while still being able to mechanically displace the phrenic nerve.

One of the most important determinants for lesion formation is the energy source used. Epicardial ablation can be performed by way of standard (nonirrigated) radiofrequency (RF) ablation, cooled (irrigated) RF ablation, bipolar ablation **(Figure 3)**, and even cryoablation. Further details relating to ablation energy sources are provided henceforth.

#### Radiofrequency ablation

Cooled irrigation has been developed to prevent heating of the catheter tip during ablation in order to allow for the delivery of sufficient RF energy. Early studies have shown that cooled or irrigated-tip RF ablation can generate epicardial lesions more effectively than standard 4-mm RF ablation.^15^ In addition, cooled-tip RF ablation creates lesions that are larger than those formed using standard RF energy. Further, irrigated ablation appears to be of particular benefit in ablating areas with overlying epicardial fat. Epicardial fat can attenuate lesion formation, especially when using standard RF energy; however, cooled-tip RF energy may overcome this barrier to promote more effective lesion formation during epicardial ablation performed over epicardial fat.^15^

#### Cryoablation

Outside of RF, cryoablation is an alternative energy source that can be used for epicardial VT ablation. Initial in vivo animal studies have demonstrated that cryoablation can produce epicardial lesions of similar sizes and depths as those on the endocardium.^16^ Follow-up studies, however, showed that epicardial cryoablation with an 8-mm-tip cryocatheter led to larger lesion volumes and diameters in infarcted myocardium in comparison with those created using a 3.5-mm irrigated RF catheter. The authors hypothesized that this was likely related to a combination of better contact through cryoadherence and a lack of warming by circulating blood.^17^ Currently, cryoablation is not widely used by tertiary care centers, as there remain, similarly to in the case of RF, concerns regarding the performance of ablation near epicardial arteries and the potential for vessel injury via neointimal proliferation.^3,18^ Additional studies are needed to directly compare RF and cryoenergy for epicardial VT ablation in order to draw more conclusions regarding safety and efficacy.

#### High-intensity ultrasound and electroporation

More recently, focused high-intensity ultrasound and electroporation have been evaluated experimentally but have yet to be adopted clinically. High-intensity ultrasound is an acoustic energy source able to deliver deep lesions through fat while sparing superficial structures.^19^ This energy source is under consideration for application in both endocardial and epicardial ablation. Electroporation is another energy modality also currently being examined for use during epicardial ablation, but it has not yet been applied clinically.^20^

### Contact force

More recently, the development of force-sensing technology has had a significant impact on the field of epicardial ablation. Increasing contact force (CF) has been shown to impact epicardial RF lesion size as well as the risk levels for steam pops, acute coronary artery injury, and phrenic nerve injury, respectively. Although epicardial fat limits lesion size, RF ablation with increasing CF can produce small myocardial RF lesions at sites of thick epicardial fat.^21^ Suboptimal catheter orientation during epicardial mapping was associated frequently with higher CF measurements. Consequently, this finding suggests that increased CF during epicardial mapping does not necessarily imply adequate myocardial contact. On the contrary, the application of higher CF epicardially can in fact redirect the ablation catheter away from the myocardium toward extracardiac structures with deleterious effects.^22^ Thus, catheter orientation is pertinent to the efficacy and safety of epicardial ablation. Another finding from the study is that bipolar signal amplitude in healthy endocardial and epicardial tissue may increase with CF values of up to 10 g but not with those beyond. As such, the best CF cutoff values for obtaining a signal amplitude of greater than 1.5 mV were determined to be 7 g in the left ventricular (LV) endocardium, 9 g in the right ventricular endocardium, and 4 g in the epicardium.^22^ These findings are consistent with those of other studies that similarly found that the optimal cutoff for CF in predicting adequate tissue contact during LV endocardial and epicardial mapping was 9 g.^23^

### Pericardial space

Another important determinant of ablation lesion formation is the pericardial environment in which epicardial ablation is being performed. An ex vivo study showed that higher irrigation flow rates yield smaller surface lesion diameters.^24^ Aside from this, there were no consistent differences in lesion depth or volume when using different flow rates. RF ablation in the presence of intrapericardial fluid led to a substantial reduction in lesion size and volume.^24^ Conclusions from this study are that ablation using reduced flow rates will result in slower intrapericardial fluid accumulation, a reduced need for pericardial drainage, and larger ablation lesions, without an increased risk of steam pops. Additional ex vivo and in vivo studies have confirmed that increased fluid in the pericardial space leads to smaller lesion formation and that a higher impedance fluid in this same space can facilitate effective delivery of RF ablation to the myocardium.^25^

### Epicardial fat

As somewhat alluded to earlier, one of the most important problems in the setting of epicardial ablation is the presence of fat, which can significantly reduce the efficacy of the RF energy. Unfortunately, anatomic locations of epicardial fat sometimes coincide with desired targets for ablation. The presence of epicardial fat interposed between an ablation catheter and underlying epicardium may result in ineffective delivery of RF energy and inadequate lesion formation,^26,27^ with prior animal studies showing that RF energy delivery can be attenuated by even a few millimeters of epicardial fat.^15^ Epicardial fat is most prominent at the base and in perivascular regions, but a recent study characterizing epicardial fat by CT in a series of patients revealed that epicardial fat often can extend to areas well beyond these segments with average thicknesses of several millimeters.^28^

Epicardial fat of more than 5 mm in thickness can mimic scar, though electrograms of real scar tend to be longer in duration with more fractionation and late potentials, while those for epicardial fat tend to have higher impedance values. Thick epicardial fat (> 5 mm) can also decrease electrogram amplitude and prevent ventricular pacing capture at high outputs.^29^ Epicardial fat is increased in patients with coronary artery disease and positively correlates with the staging of cardiomyopathy.^30^

### Epicardial coronary vessels

Epicardial ablation can be unsuccessful due to the presence of adjacent coronary arteries to the desired ablation target. The current guidelines recommend a distance of 5 mm to be maintained between the ablation catheter and coronary arteries in order to avoid injury to the vessel.^10^ In a study of more than 300 ablation procedures, epicardial ablation was aborted in 13% of cases due to proximity to the coronary arteries during RF ablation.^31^ In this particular study, it was found that epicardial ablation was deemed unfeasible for many cases in which the origin of the ventricular arrhythmia was at the LV summit region, mainly due to coronary artery proximity. Previous studies have similarly noted difficulty when ablating along both the anterior wall of the LV and the LV summit region due to the close proximity of coronary arteries.^32,33^

### Modulators of energy source

Irrigation of the catheter tip leads to cooling of the catheter tip–tissue interface, thereby allowing for greater power delivery. Catheter irrigation has been standardly achieved with normal saline irrigation. However, the ionic concentration and lower impedance of normal saline can divert RF energy away, thus decreasing our ability to create an effective lesion. Decreasing the ionic concentration of the irrigant by using a fluid such as half-normal saline can increase the surrounding impedance and thus decrease the loss of RF to the surrounding environment, allowing for greater RF delivery **(Figure 1B)**.^25,34^ With regard to environmental impedance, this may be of greater importance in the epicardium, given the presence of intrapericardial fluid and epicardial fat.

Bipolar ablation has been used previously to ablate deep myocardial VTs.^35,36^ When the arrhythmogenic focus involves nonseptal myocardium, epicardial access may be necessary to allow for the establishment of a bipolar circuit from the endocardium to epicardium, thereby sandwiching the focus **(Figure 3)**.

## Conclusion

In the last two decades, breakthroughs in technology with the advent of CF and other alternative energy sources have allowed us to perform more successful epicardial ablation procedures. Understanding barriers to successful ablation (e.g., the thickness of epicardial fat) will help us deliver even more effective ablation lesions to the tissue while minimizing potential complications. A full understanding of how each of these factors plays a role in lesion formation is essential for successful epicardial ablation.
